# Identification of *KRAS*^*G12C*^ Mutations in Circulating Tumor DNA in Patients With Cancer

**DOI:** 10.1200/PO.21.00547

**Published:** 2022-07-21

**Authors:** Kyaw Z. Thein, Amadeo B. Biter, Kimberly C. Banks, Andrew W. Duda, Jennifer Saam, Jason Roszik, Filip Janku, Ferdinandos Skoulidis, John V. Heymach, Scott Kopetz, Funda Meric-Bernstam, David S. Hong

**Affiliations:** ^1^ Department of Investigational Cancer Therapeutics, The University of Texas MD Anderson Cancer Center, Houston, TX; ^2^ Division of Hematology and Medical Oncology, Oregon Health and Science University/Knight Cancer Institute, Portland, OR; ^3^ Guardant Health, Inc., Redwood City, CA; ^4^ Department of Melanoma Medical Oncology, The University of Texas MD Anderson Cancer Center, Houston, TX; ^5^ Department of Thoracic-Head & Neck Medical Oncology, The University of Texas MD Anderson Cancer Center, Houston, TX; ^6^ Department of Gastrointestinal Medical Oncology, The University of Texas MD Anderson Cancer Center, Houston, TX

## Abstract

*KRAS* is the most mutated proto-oncogene that has been identified in cancer, and treatment of patients with *KRAS* mutations remains an arduous challenge. Recently, *KRAS*^*G12C*^ mutation has attracted special interest because it is now considered potentially druggable with recently developed covalent small-molecule *KRAS*^*G12C*^ inhibitors. Nevertheless, to date, there have been no large-scale analyses of liquid biopsy that include testing for *KRAS*^*G12C*^. Here, we performed a comprehensive analysis of *KRAS*^*G12C*^ mutations in multiple cancer types, as detected by circulating tumor DNA.

**METHODS:**

We conducted a 5-year retrospective review of *KRAS*^*G12C*^ mutations in patients with cancer who had undergone Guardant360 testing between July 1, 2014, and June 30, 2019; our study included treatment-naive and previously treated patients with metastatic solid tumors.

**RESULTS:**

*KRAS*^*G12C*^ mutations were identified in 2,985 of 80,911 patients (3.7%), across > 40 tumor types. *KRAS*^*G12C*^ mutations were detected most frequently in patients with nonsquamous non–small-cell lung cancer (NSCLC; 7.5%), NSCLC of all subtypes (6.9%), cancer of unknown primary (4.1%), colorectal cancer (3.5%), squamous NSCLC (2.0%), pulmonary neuroendocrine tumors (1.9%), and pancreatic ductal adenocarcinoma (1.2%) and cholangiocarcinoma (1.2%). *KRAS*^*G12C*^ mutations were predominantly clonal (clonality > 0.9%) in patients with lung adenocarcinoma, non-NSCLC, cancer of unknown primary, NSCLC, and pancreatic ductal adenocarcinoma, and patients with colorectal cancer and breast cancer had bimodal distribution of clonal and subclonal *KRAS*^*G12C*^ mutations.

**CONCLUSION:**

Our study demonstrates the feasibility of using circulating tumor DNA to identify *KRAS*^*G12C*^ mutations across solid tumors; the highest detection rate was in lung cancer, as previously reported in the literature.

## INTRODUCTION

The role of the rat sarcoma viral oncogene (*RAS*) in tumorigenesis was discovered approximately 3 decades ago.^1-3^ *RAS* activates and triggers downstream intracellular signaling cascades, including the mitogen-activated protein kinase, signal transducer and activator of transcription, and phosphoinositide 3-kinase pathways.^4,5^ Kirsten RAS (*KRAS*), one of three *RAS* isoforms, is the most commonly mutated proto-oncogene that has been identified in cancer.^1,2,6^ Despite its frequency and decades of research, the treatment of patients with *KRAS* mutations still remains an arduous challenge. However, the *KRAS*^*G12C*^ mutation has recently attracted special interest since the development of covalent small-molecule *KRAS*^*G12C*^ inhibitors.^7-11^

**CONTEXT**

**Key Objective**
This retrospective study examined *KRAS*^*G12C*^ mutations using circulating tumor DNA (ctDNA) across solid tumors of patients who were tested by Guardant360 assay. To our knowledge, this is the first large-scale study to demonstrate the feasibility of using ctDNA.
**Knowledge Generated**
3.7% of 80,911 patients across > 40 tumor types had *KRAS*^*G12C*^ mutations identified in ctDNA. *KRAS*^*G12C*^ mutations were predominantly clonal in patients with lung cancer, cancer of unknown primary, and pancreatic ductal adenocarcinoma, and patients with colorectal cancer and breast cancer had bimodal distribution of clonal and subclonal *KRAS*^*G12C*^ mutations. We found very high positive predictive value between tissue and liquid biopsies performed within 6 months of each other while positive predictive value was lower at 77%, between tests conducted > 6 months apart. Discordant rates differed by tumor type and clonality.
**Relevance**
These findings demonstrate the feasibility of using ctDNA to identify *KRAS*^*G12C*^ mutations across solid tumors. Clonality information from ctDNA-based genotyping may provide insights into the clinical efficacy of targeting *KRAS*^*G12C*^.

Advances in next-generation sequencing have revealed the complex genomic landscape of various cancers and have uncovered more genetic alterations and novel genomic drug targets than have hotspot mutation tests.^12,13^ Additionally, using circulating tumor DNA (ctDNA) in plasma has the potential to overcome the limitations associated with tissue biopsies, including complications from invasive procedures, incomplete genotyping caused by insufficient tissue quantity or quality, and the inaccessibility of some metastatic lesions.^14,15^ ctDNA is cell-free DNA that sheds into the bloodstream not only from the main tumor site but also from metastatic lesions. Thus, ctDNA hypothetically represents an anatomically unbiased sample that demonstrates both intertumoral and intratumoral heterogeneity.^12,13,16-18^ Studies have shown the feasibility and clinical utility of large-scale liquid biopsies in some types of cancer, such as lung cancer and colorectal cancer (CRC).^12,13^

We performed a comprehensive analysis of *KRAS*^*G12C*^ mutations in 80,911 patients with cancer, including detection rate and clonality by cancer type, co-occurring mutations in lung cancer and CRC, and concordance of ctDNA with tissue, using ctDNA. To our knowledge, this is the first large-scale study to demonstrate the feasibility of using ctDNA to detect *KRAS*^*G12C*^ in solid tumors.

## METHODS

### Study Design

We performed a retrospective review of consecutive ctDNA results from patients who had undergone clinical Guardant360 testing between July 1, 2014, and June 30, 2019. Both treatment-naive and previously treated patients with metastatic solid tumors were included in the analysis. This retrospective review was approved by Institutional Review Board. Data were deidentified and analyzed in accordance with the Institutional Review Board guidelines. The clonality of *KRAS*^*G12C*^, defined as the variant allele fraction of the *KRAS*^*G12C*^/maximum somatic allele fraction in the sample, was analyzed by cancer type, and mutation co-occurrence landscape was interrogated for cancer types with > 100 unique patients, specifically CRC and lung cancer. We further reviewed the subset of cases with available tissue results to identify the concordance of ctDNA with tissue. Using institutional records, we also obtained basic demographic and outcome information on patients from The University of Texas MD Anderson Cancer Center (Houston, TX).

### ctDNA Analysis

The Guardant360 assay, which has been certified by Clinical Laboratory Improvement Amendments, the College of American Pathology, and the state of New York, was performed on plasma as previously described.^19,20^ Over the time frame of this analysis, multiple iterations of the test were used, and all iterations of the test analyzed *KRAS*, *APC*, *TP53m*, and *EGFR*. The Guardant360 assay can analyze point mutations in 54-74 genes, copy number amplifications in up to 18 genes, and fusions in up to six genes.

Samples with no somatic alterations detected were excluded. Patients with more than one test were counted only once when calculating the *KRAS*^*G12C*^ detection rate. We also reviewed cases with tissue testing for *KRAS* mutations. We identified a subset of cases, comprising patients from MD Anderson, and reviewed their clinical and demographic data.

### Statistical Analysis

Descriptive statistics were used in this analysis.

### Ethical Approval and Consent to Participate

All individuals provided consent for clinical testing, and testing data were deidentified for analysis. Institutional Review Board approval was obtained for this study, and a waiver of informed consent was obtained because of our study's retrospective nature.

## RESULTS

### *KRAS*^*G12C*^ Detection by Cancer Type

**FIG 1.**
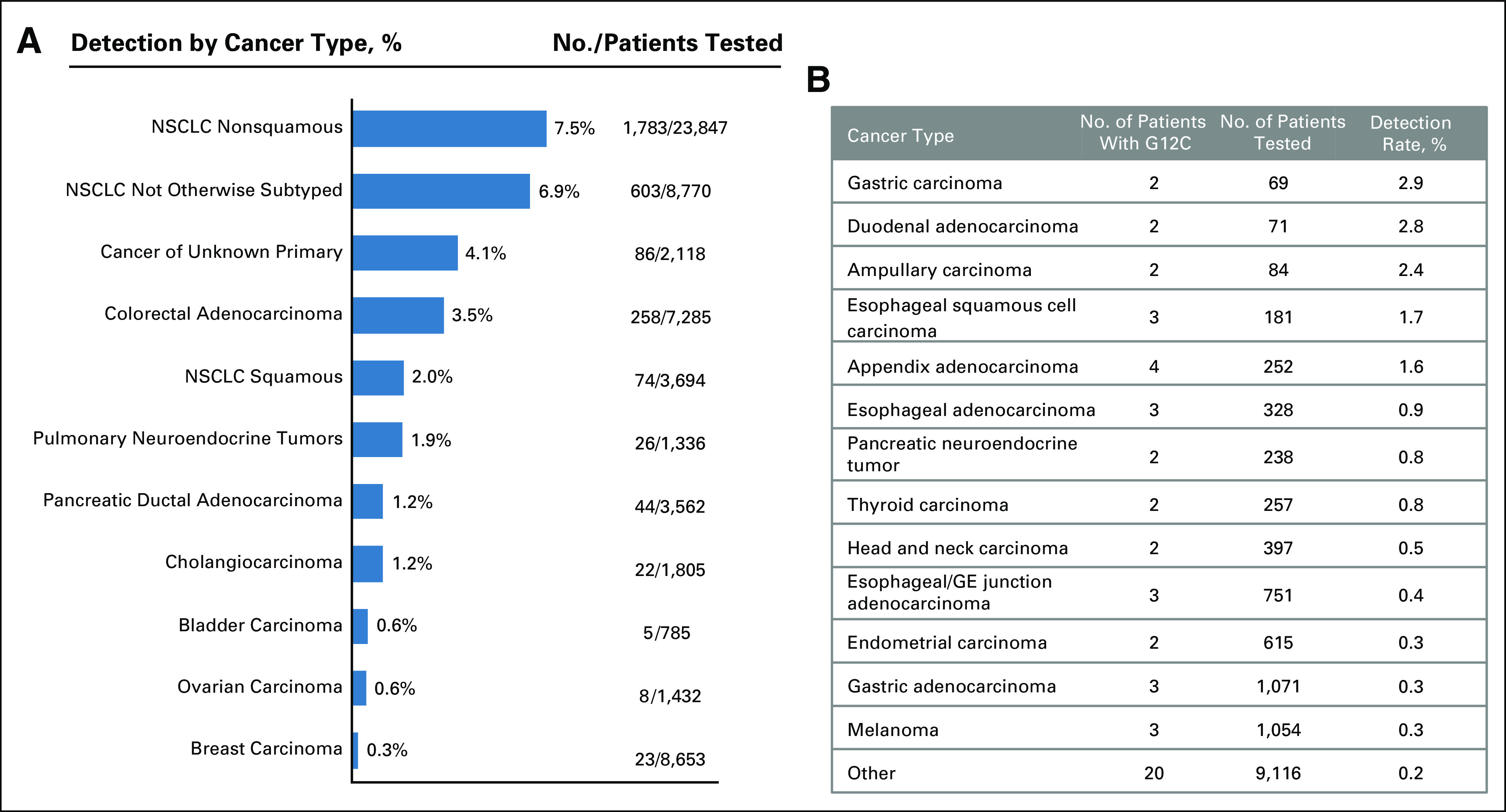
*KRA^G12C^* mutations by cancer type in the Guardant360 database. (A) Bar graph limited to cancer types with five or more unique patients with *KRAS*^*G12C*^ mutations, with percentages calculated by subtype. (B) Table captures other subtypes with < five patients with *KRAS*^*G12C*^ mutations, including subtypes with just one case (other). CUP, cancer of unknown primary; GE, gastroesophageal; No., number; NSCLC, non–small-cell lung cancer.

**FIG A1.**
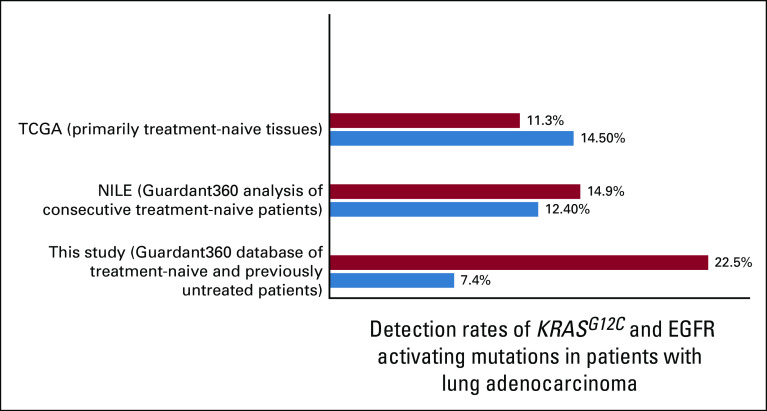
*KRAS^G12C^*- and EGFR-activating mutations in patients and tissues with lung adenocarcinoma in our study, NILE, and TCGA. Blue color highlights *KRAS*^*G12C*^ mutations and red color highlights EGFR-activating mutations. Current study, Guardant360 database of treatment-naive and previously untreated patients; NILE study, Noninvasive versus Invasive Lung Evaluation, Guardant360 analysis of consecutive treatment-naive patients; TCGA study, The Cancer Gene Atlas, primarily treatment-naive tissues.

We identified 80,911 unique patients whose ctDNA was tested using the Guardant360 assay between July 1, 2014, and June 30, 2019. A *KRAS*^*G12C*^ mutation was identified in 2,985 patients (3.7%) across > 40 tumor types, most frequently in patients with non–small-cell lung cancer (NSCLC; 7.5%), followed by other lung cancers (6.9%), cancer of unknown primary (CUP; 4.1%), CRC (3.5%), squamous NSCLC (2.0%), pancreatic ductal adenocarcinoma (PDAC; 1.2%), cholangiocarcinoma (1.2%), bladder carcinoma, and other solid tumor types (Fig 1). Although these relative frequencies mirror those seen in tissues across tumor types, the absolute numbers differ, particularly for NSCLC. This is most likely due to not only the inclusion of both treatment-naive and previously treated patients in the analysis but also a bias created by liquid biopsy ordering patterns (eg, clinicians order liquid biopsies at disease progression for patients with *EGFR-*mutant NSCLC more frequently than for patients undergoing nontargeted therapies). To confirm the impact of ordering bias, we compared the detection rates of *KRAS*^*G12C*^- and *EGFR*-activating mutations in lung adenocarcinoma among patients included in this study, newly diagnosed patients from the Noninvasive versus Invasive Lung Evaluation (NILE) study (14), and primarily treatment-naive tissues from The Cancer Genome Atlas (TCGA; 22; Appendix Fig A1). We found that the detection rates of *KRAS*^*G12C*^ in patients with lung adenocarcinoma was 7.5% in our study, 13.1% in the NILE study (same ctDNA assay), and 14.5% in TCGA. In contrast, the frequency of *EGFR*-activating mutations was 23.2% in our study, 14.9% in the NILE study, and 11.3% in TCGA; this *EGFR* mutation distribution compared with *KRAS* mutation distribution is consistent with the hypothesized ordering bias.

### *KRAS*^*G12C*^ Clonality by Cancer Type

**FIG 2.**
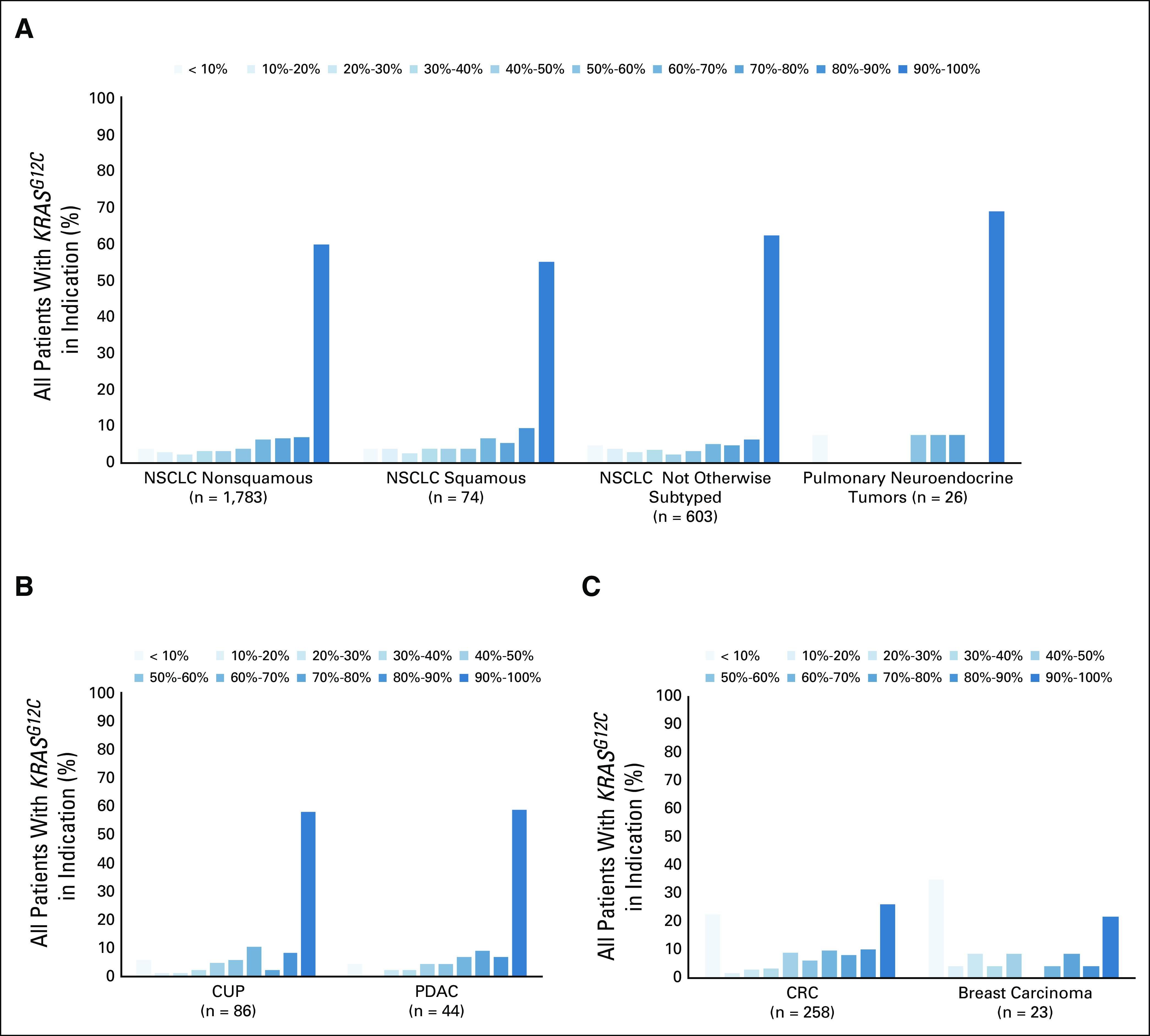
*KRAS^G12C^* clonality among (A) lung cancer subtypes, where the majority of *KRAS^G12C^* mutations are clonal; (B) other cancer types with primarily clonal *KRAS^G12C^*; and (C) cancers with bimodal distribution of *KRAS^G12C^* clonality. CLIA, Clinical Laboratory Improvement Amendments; CRC, colorectal cancer; CUP, cancer of unknown primary; NSCLC, non–small-cell lung cancer; PDAC, pancreatic ductal adenocarcinoma.

*KRAS*^*G12C*^ clonality by cancer type was analyzed; clonality was defined as the variant allele fraction/maximum somatic allele fraction in the sample. The *KRAS*^*G12C*^ mutation was found to be clonal (defined as clonality > 0.9) in most patients with lung adenocarcinoma, NSCLC, CUP, SCLC, and PDAC. In comparison, clonality was bimodally distributed in patients with CRC and breast cancer (Fig 2).

### Landscape of Co-Occurring Mutations in *KRAS*^*G12C*^-Mutant Lung Cancers and CRC

**FIG 3.**
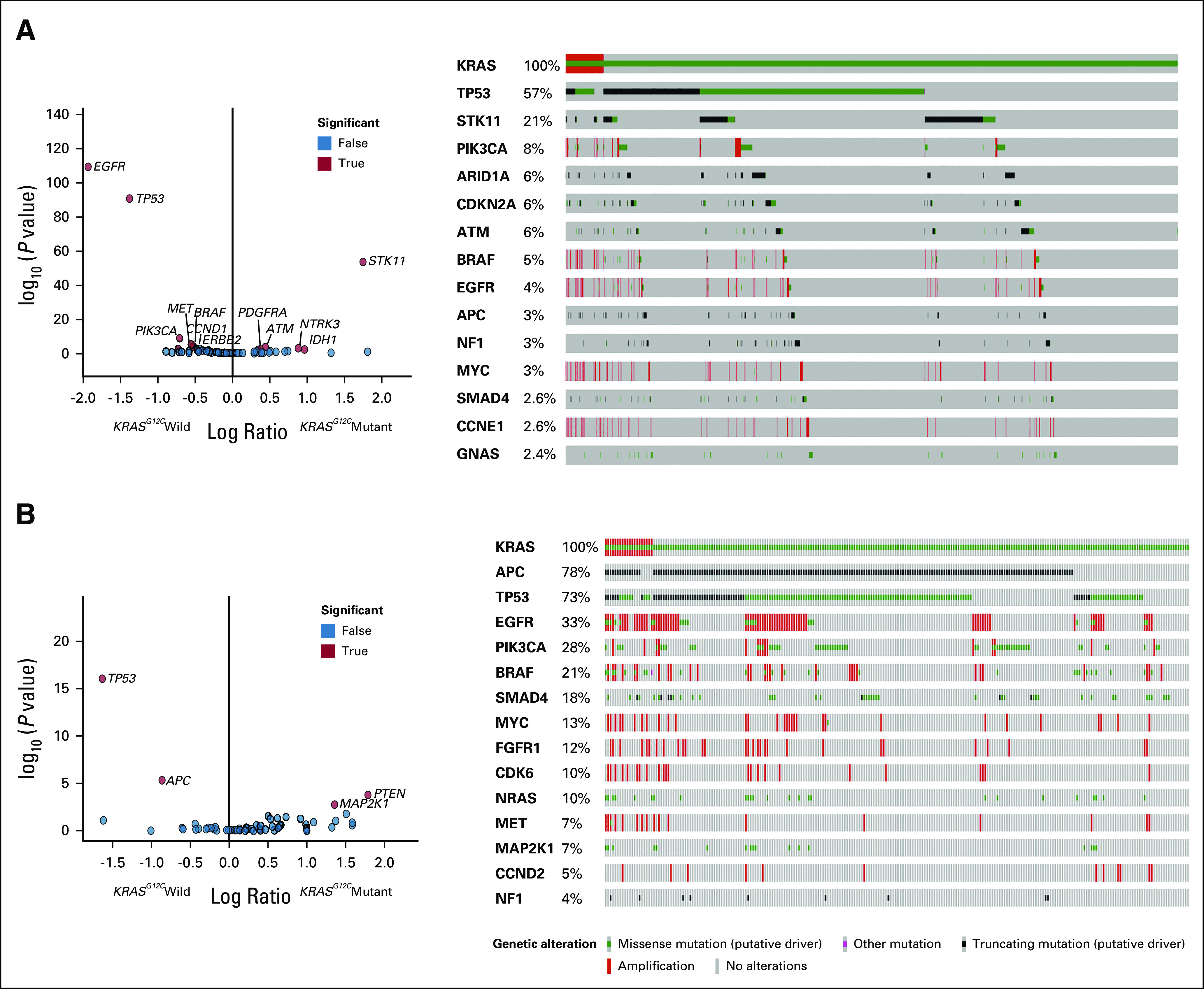
Volcano plot and OncoPrint of co-occurring mutations in (A) lung and (B) colorectal cancer.

As seen on the volcano plot and OncoPrint in Figure 3A, *EGFR* and *TP53* were found to be enriched in the *KRAS*^*G12C*^ wild-type lung cancer while *STK11* was a more common co-occurring mutation in *KRAS*^*G12C*^-mutant lung cancer. Similarly, *TP53* and *APC* were enriched in *KRAS*^*G12C*^ wild-type CRC while *MAP2K1* and *PTEN* were commonly coaltered aberrations in *KRAS*^*G12C*^-mutant CRC (Fig 3B).

### Comparison of ctDNA With Tissue Biopsy in *KRAS*^*G12C*^-Mutant Cancers

**FIG 4.**
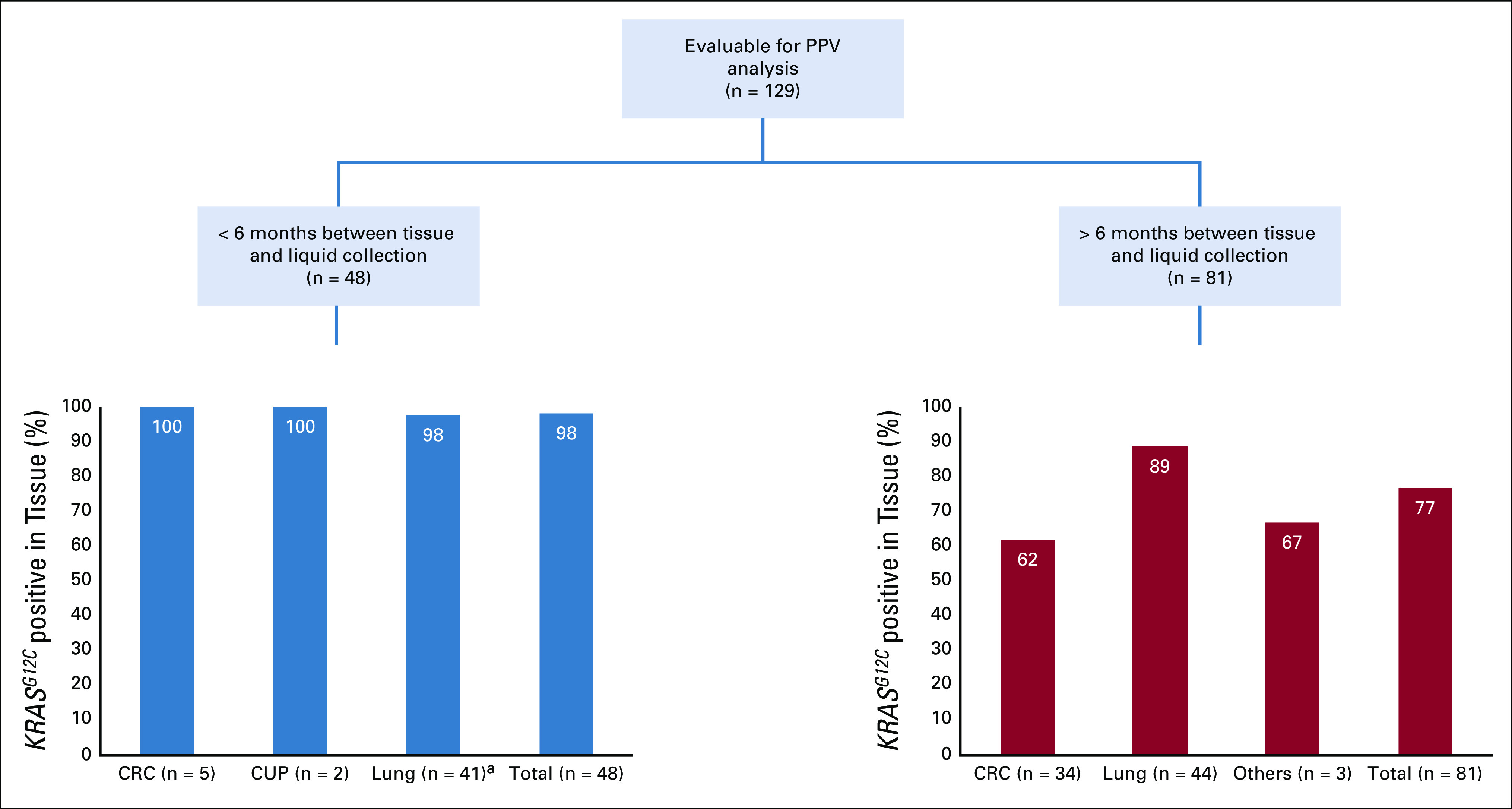
Evaluable cohort of patients with known *KRAS* tissue testing results. ^a^Pathology report stated sparse tumor presents in cell block for a patient with lung cancer who had *KRAS*^*G12C*^-negative tissue but was tested positive for *KRAS*^*G12C*^ by Guardant360. CRC, colorectal cancer; CUP, cancer of unknown primary; PPV, positive predictive value.

Of the 2,985 patients with *KRAS*^*G12C*^ identified by ctDNA, 151 had documented previous tissue testing (Table 1). Twenty-two patients did not have tissue genomic studies completed because of insufficient tissue. Hence, 129 patients had both tissue and ctDNA results available for concordance analysis (84 NSCLC, 39 CRC, and eight others as described in Table 1). The cohort was divided into two groups on the basis of the elapsed time between the tissue biopsy and ctDNA analysis (Fig 4): Time synchronous was defined as < 6 months between tissue biopsy and ctDNA analysis (48 patients); time asynchronous was defined as > 6 months between tissue and ctDNA analysis (81 patients).

**TABLE 1.**
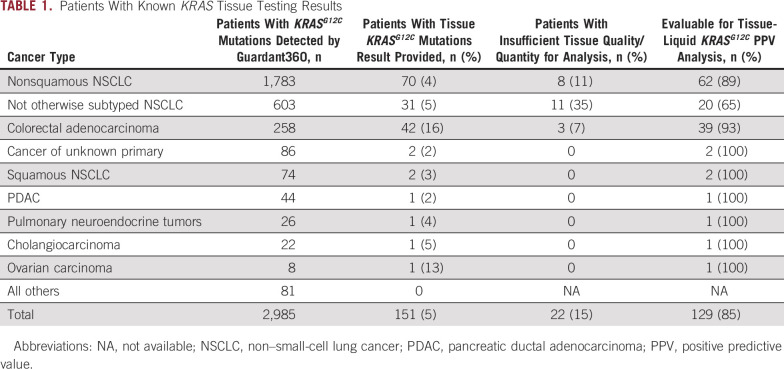
Patients With Known *KRAS* Tissue Testing Results

High concordance (98%, 47 of 48) was found in the time synchronous group while the time asynchronous group showed greater discordance (11% in NSCLC, 38% in CRC, and 33% overall; Table 2). In the time synchronous group, concordance between ctDNA analysis and tissue biopsy was 100% for patients with CRC and 98% for patients with lung cancer. Of note, the one discordant sample occurred in a lung cancer case whose negative tissue report noted that sparse tissue was present in the cell block which likely explains the discordant results. Tissue from 34 patients with CRC, 44 patients with lung cancer, and one each with cholangiocarcinoma, ovarian cancer, and pancreatic cancer was tested > 6 months before ctDNA testing. In these patients, *KRAS*^*G12C*^ had not been detected in tissue for 13 with CRC, 5 with lung cancer, and 1 with cholangiocarcinoma. Overall concordance (77%) was lower in these patients than in those with tests more than 6 months apart. Discordance was highest for patients with CRC, at 38%, compared with that for those with lung cancer, at 11%.

**TABLE 2.**
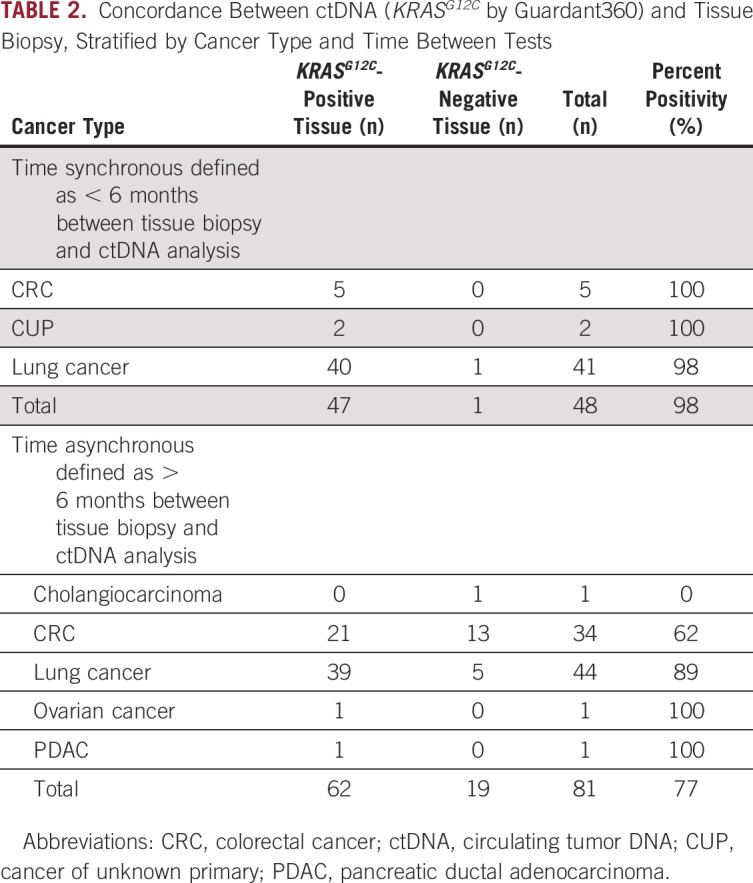
Concordance Between ctDNA (*KRAS*^*G12C*^ by Guardant360) and Tissue Biopsy, Stratified by Cancer Type and Time Between Tests

**FIG A2.**
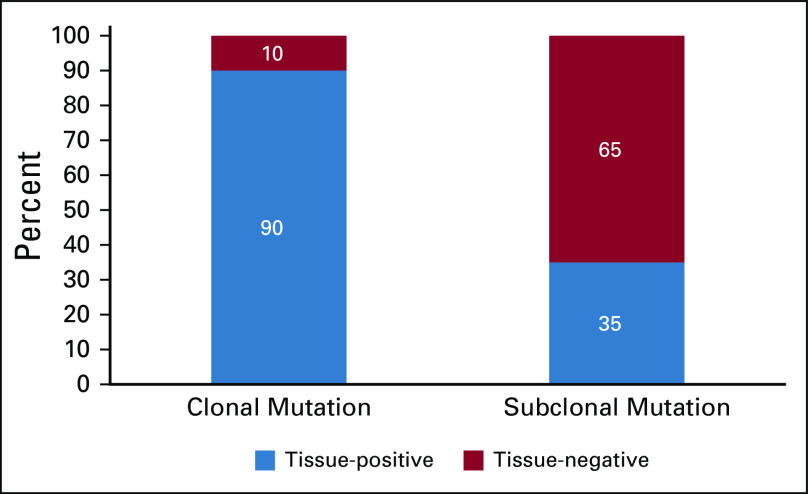
*KRAS*^*G12C*^ positivity in tissue, stratified by clonality of *KRAS*^*G12C*^ in ctDNA, as assessed in tissues and ctDNA collected > 6 months apart. ctDNA, circulating tumor DNA.

Additional concordance analysis was performed on the basis of clonality; 104 patients had clonal *KRAS*^*G12C*^ and 25 had subclonal *KRAS*^*G12C*^ by ctDNA. The discordance was only present in patients who underwent biopsy at least 6 months before ctDNA (Appendix Fig A2). The overall concordance was 90% in the clonal group, and *KRAS*^*G12C*^ was not detected in tissue in 100%, 5%, and 11% of patients with cholangiocarcinoma, CRC, and lung cancer, respectively (Appendix Table A1). Among the 20 patients with a subclonal mutation by ctDNA who were tested at least 6 months apart, *KRAS*^*G12C*^ was detected in tissue in only 35% (Appendix Table A1). In particular, 12 of 13 patients with CRC and one of seven with lung cancer had discordant results.

### Clinical Description of Discordant Clonal *KRAS*^*G12C*^ Mutations (> 6 months between tissue and liquid biopsies)

We reviewed the clinical histories of the subset of patients who were tested at MD Anderson (Appendix Table A2). The highest discordance between tissue biopsy and ctDNA analyses was among patients with CRC: 10 of 11 had clinical histories that suggested that *KRAS*^*G12C*^ arose as a result of treatment resistance. In eight patients with CRC with repeat liquid biopsies, one additional mutation in the mitogen-activated protein kinase/extracellular signal–regulated kinases (MAPK/ERK or RAF/MEK/ERK) pathway was detected in addition to *KRAS*^*G12C*^, consistent with acquired resistance to monoclonal antibody therapy. In one patient with CRC who had tissue and liquid biopsies approximately 5.3 years apart, only *KRAS*^*G12C*^ with clonality between 30% and 40% was detected upon repeat ctDNA testing, and there was no other obvious mutation that could drive acquired resistance to monoclonal antibodies.

A patient with cholangiocarcinoma experienced disease progression on TAS-120 and underwent liquid biopsy approximately 5 years after initial tissue testing that showed an *FGFR* mutation. Multiple likely resistance mutations were detected in the ctDNA, including *KRAS*^*G12C*^ with 58% clonality. All four patients with lung cancer had clonality between 90% and 100%. *KRAS*^*G12C*^ was not identified in tissues collected from two patients who underwent tissue and liquid biopsies 10 months and 2 years apart. We hypothesize that *KRAS*^*G12C*^ was not identified in these tissues because of insufficient tissue quantity, given that no other biomarkers were identified. *KRAS*^*G12C*^ was also not detected by liquid biopsy in another patient who had been diagnosed with stage I lung cancer by tissue biopsy approximately 6 years previously. In another patient with lung adenocarcinoma who was *EGFR*-positive and experienced disease progression on osimertinib, ctDNA showed a subclonal *KRAS*^*G12C*^ mutation that was not detected on prior tissue biopsy, consistent with acquired resistance to *EGFR* inhibitors, as previously demonstrated.^21^

## DISCUSSION

The growing utilization of next-generation sequencing has resulted in advanced precision oncology: Biomarker testing has led to the development of many novel therapeutics and combinatorial regimens. Indeed, next-generation sequencing can be used in newly diagnosed patients to inform therapy decisions and at disease progression to monitor mechanisms of resistance.

*KRAS* is the most frequently mutated proto-oncogene, with most (approximately 80%) *KRAS* mutations being point mutations at codon 12.^22,23^ *KRAS* mutations have been historically considered to be undruggable, but the discovery by Ostrem et al^24,25^ of small molecules that covalently bind the shallow pocket between switches I and II in *KRAS*^*G12C*^ has challenged this belief. Novel *KRAS*^*G12C*^ inhibitors, such as AMG510 and MRTX849, were shown to be clinically active securing the recent US Food and Drug Administration approval of AMG510 in patients with *KRAS*^*G12C*^-mutated metastatic NSCLC, boosting the need for robust genotyping for this marker in oncology practice.^7-11,26^ Nevertheless, to date, there have been no large-scale analyses of liquid biopsy profiles that include testing for *KRAS*^*G12C*^ across solid tumor types.

In our study, 3.7% of 80,911 patients across > 40 tumor types had *KRAS*^*G12C*^ mutations identified in ctDNA via routine testing at a large diagnostic laboratory. *KRAS*^*G12C*^ was detected most frequently in patients with lung cancer, followed by CUP and CRC. *KRAS* has long been known to be a driver in lung cancer and CRC as well as a well-established acquired resistance mechanism to targeted therapies in lung, CRC, and other rare cancer types. *KRAS*^*G12C*^ was identified in rarer and hard-to-treat cancers such as PDAC, ovarian carcinoma, and cholangiocarcinoma. We found that *KRAS*^*G12C*^ was clonal (clonality > 0.9) in cancer types except for patients with CRC and breast cancer, in which clonality was bimodal, suggesting that *KRAS* is a more common resistance mechanism in these tumor types. *EGFR* and *TP53* mutations were enriched in *KRAS*^*G12C*^ wild-type lung cancer while *STK11* was a more common co-occurring mutation in patients with *KRAS*^*G12C*^. The literature shows the same result of *STK11* mutations co-occurring with *KRAS*^*G12C*^ at a high frequency and provides confidence in the ability of liquid biopsy to provide the same results as tissue assays.^27^ In contrast, *TP53* and *APC* mutations were enriched in *KRAS*^*G12C*^ wild-type CRC, and *MAP2K1* and *PTEN* co-occurred more frequently in patients with *KRAS*^*G12C*^-mutant CRC.

We demonstrated that in 98% of cases in which tissue and liquid biopsies were performed within 6 months, *KRAS*^*G12C*^ was detected by both modalities; the detection rate in the tissue analysis was 100% in CRC and 98% in lung cancer. This high concordance between tissue and ctDNA when performed close together in time is consistent with the results of previous studies. In patients tested by tissue and liquid biopsy > 6 months apart, the detection rate in tissue was lower at 77%. Concordance varied by tumor type and clonality, at 62% and 89% in CRC and lung cancer tissue, respectively. Concordance was 90% in patients with clonal *KRAS*^*G12C*^ but only 35% in patients with sub clonal *KRAS*^*G12C*^. Most patients with CRC for whom *KRAS*^*G12C*^ was not detected on prior tissue biopsy likely had acquired resistance to targeted therapy, regardless of clonality. This pattern suggests that the most likely explanation for why the *KRAS*^*G12C*^ mutation was not detected in tissue but was detected in ctDNA analysis was due to a resistance mechanism that arose over time and was, therefore, subclonal in liquid and not previously detected in the pretreatment tissue. Clinical cases are consistent with this explanation.

With several more targeted cancer therapies becoming available, efficient and easy genotyping at diagnosis is critical. Moreover, upon disease progression, given the demonstrated role and emergence of *KRAS^G12C^* mutations, both as primary and acquired resistance mechanisms, real-time genomic insights with ctDNA analysis can provide to inform further line of therapy. This is particularly urgent in lung cancer, where mutations in EGFR, ALK, ROS, RET, METex14 skipping, BRAF, and KRAS should be identified before initial treatment. Moreover, the limitations of tissue, particularly for lung cancer, with small specimens and multiple biomarkers to test make the value of well-validated ctDNA assay option necessary to provide comprehensive patient care.

Our study has many strengths. To our knowledge, it is the first and largest analysis *KRAS*^*G12C*^ mutations using ctDNA across solid tumors, with more than 80,000 unique patients with metastatic solid tumors who were tested by the Clinical Laboratory Improvement Amendments–certified, College of American Pathology–accredited, and recently US Food and Drug Administration–approved Guardant360 assay. In addition, the review of *KRAS*^*G12C*^ clonality by cancer type and assessment of patterns of co-occurring mutations in lung and CRC was possible given the insights ctDNA analysis provides because it captures intratumor and intertumor heterogeneity and is quantitative providing insights into clonality. Furthermore, we compared the mutational landscape of ctDNA with that of tissue biopsy which confirmed high positive predictive value for time synchronous samples suggesting that liquid or tissue can be used for up-front profiling, and the greater discordance seen in time asynchronous cases reinforced the importance of evolving genomic landscapes under treatment pressure and value ctDNA provides. Finally, we analyzed discordance between tissue and liquid biopsy in the context of clonality and elapsed time between tests.

One key limitation of our study is that the Guardant360 database includes both treatment-naive and previously treated patients without the necessary details to analyze separately limiting ability to compare detection rates from this study with prevalence rates previously published. Whereas, the first-line NILE study and TCGA only include treatment-naive patients. There may also be bias created by ordering patterns for liquid biopsies as the relative detection rates across tumor types mirror those seen in tissue, yet absolute numbers differ.

In conclusion, our study demonstrates the feasibility of using ctDNA to identify *KRAS*^*G12C*^ mutations across solid tumors, with the highest detection rate in lung cancer as previously noted in the literature. We also found very high positive predictive value between tissue and liquid biopsies performed within 6 months of each other while the positive predictive value was lower at 77%, between tests conducted > 6 months apart. Discordant rates differed by tumor type and clonality. Indeed, clonality information from ctDNA-based genotyping may provide insights into the clinical efficacy of targeting *KRAS*^*G12C*^ in different tumor types.

## Figures and Tables

**TABLE A1.**
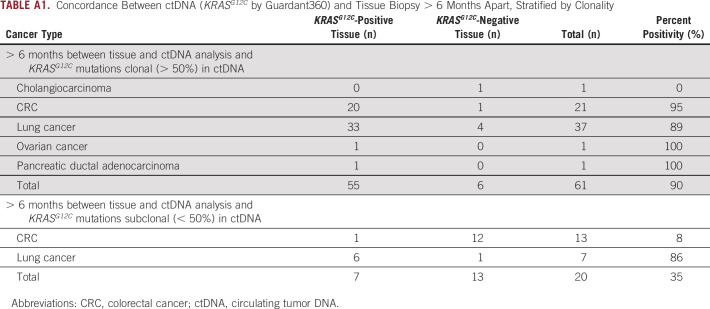
Concordance Between ctDNA (*KRAS*^*G12C*^ by Guardant360) and Tissue Biopsy > 6 Months Apart, Stratified by Clonality

**TABLE A2.**
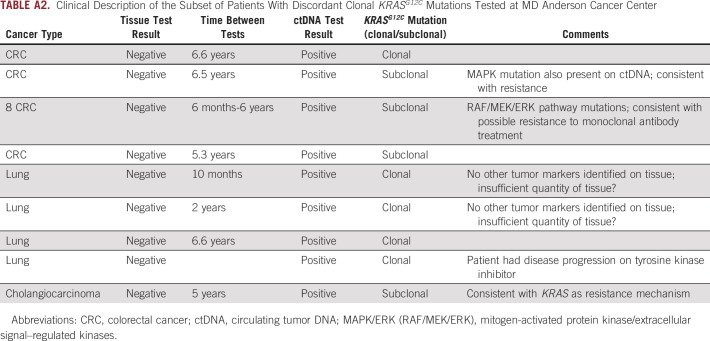
Clinical Description of the Subset of Patients With Discordant Clonal *KRAS*^*G12C*^ Mutations Tested at MD Anderson Cancer Center
